# *Nutrition & Metabolism* Classics: a disconnect between highly cited and highly accessed articles

**DOI:** 10.1186/1743-7075-11-13

**Published:** 2014-03-19

**Authors:** M Mahmood Hussain, Lucy Abel, Ahmed Bakillah

**Affiliations:** 1 Department of Cell Biology, SUNY Downstate Medical Center, 450 Clarkson Ave, Brooklyn, NY 11203, USA; 2 BioMed Central, 236 Gray’s Inn Road, London WC1X 8HB, UK

## Abstract

*Nutrition & Metabolism* has grown considerably in the ten years since its first article was published. To see how papers published in the journal had an impact we have identified some of the most popular articles in order to measure their influence, observe which fields are important to our readers, and try to explain what made these articles *Nutrition & Metabolism* “Classics”.

## 

To find out which of the published articles had a significant impact on the scientific community, we first looked at the articles that were highly cited. We used two sources to obtain citation numbers, Thomson Reuters Web of Knowledge and Google Scholar. Thomson Reuters indexes only selected journals in Web of Knowledge and provides Impact Factors via its annual Journal Citation Report, which has included *Nutrition & Metabolism* since 2006. In contrast, Google Scholar has no editorial restrictions on indexing and includes all articles published since the journal launched in 2004, therefore giving a more comprehensive and non-selective overview of citations. We selected the top 10 highly cited articles in each indexing service (Tables 1 &2). It is interesting to note that the top 10 highly cited articles, with two exceptions, were different in these two databases. A reason is that nine of the eleven highly cited articles in Google Scholar are from 2004–2005. Therefore, for an appropriate comparison, we identified highly cited articles in Google Scholar published since 2006 (Table 3). By comparing the number of citations for the same article across both indexing services we have been able to identify several articles that appear to have been cited almost exclusively by articles in journals that are themselves indexed in the Web of Knowledge. Given the stringent criteria required to be indexed by Thomson Reuters, this may well be a sign of quality. Unsurprisingly, most articles receive a greater number of citations in Google Scholar. An interesting feature of this analysis was that 60% of the highly cited papers in Thomas Reuters were research articles. In contrast, 50% of the highly cited papers according to Google Scholar were review articles. In general, review articles are the most highly cited articles in a journal, so we were pleased to note that a significant number of our research articles topped the rankings in both these indexing services. Overall, the variability in the list of high-impact articles identified by these two services highlights the inconsistency of different indexing services, and provides further evidence that the citation indexing is an imprecise measure of article quality and its impact on research.

**Table 1:** Highly cited articles in Google Scholar (retrieved 30 January 2014)

**Title**	**Year published**	**Article type**	**Citations**	**Link**
Fructose, insulin resistance, and metabolic dyslipidemia	2005	Review	400	http://www.nutritionandmetabolism.com/content/2/1/5
Uric acid: a new look at an old risk marker for cardiovascular disease, metabolic syndrome, and type 2 diabetes mellitus: The urate redox shuttle	2004	Review	299	http://www.nutritionandmetabolism.com/content/1/1/10
Chocolate and prevention of cardiovascular disease: a systematic review	2006	Review	206	http://www.nutritionandmetabolism.com/content/3/1/2
Cancer as a metabolic disease	2010	Review	159	http://www.nutritionandmetabolism.com/content/7/1/7
The role of glucocorticoid action in the pathophysiology of the metabolic syndrome	2005	Review	142	http://www.nutritionandmetabolism.com/content/2/1/3/
Effects of curcumin on retinal oxidative stress and inflammation in diabetes	2007	Research	138	http://www.nutritionandmetabolism.com/content/4/1/8
Dietary protein intake and renal function	2005	Review	128	http://www.nutritionandmetabolism.com/content/2/1/25
Diet induced thermogenesis	2004	Review	127	http://www.nutritionandmetabolism.com/content/1/1/5
Carbohydrate restriction improves the features of metabolic syndrome. Metabolic syndrome may be defined by the response to carbohydrate restriction	2005	Review	121	http://www.nutritionandmetabolism.com/content/2/1/31
Role of a critical visceral adipose tissue threshold (CVATT) in metabolic syndrome: implications for controlling dietary carbohydrates: a review	2004	Review	116	http://www.nutritionandmetabolism.com/content/1/1/12

**Table 2:** Highly cited articles according to Thomson Reuters Web of Knowledge (retrieved 16 January 2014)

**Title**	**Year published**	**Article type**	**Citations**	**Link**
Chocolate and prevention of cardiovascular disease: a systematic review	2006	Review	79	http://www.nutritionandmetabolism.com/content/3/1/2
AICAR inhibits adipocyte differentiation in 3T3L1 and restores metabolic alterations in diet-induced obesity mice model	2006	Research	62	http://www.nutritionandmetabolism.com/content/3/1/31
Cancer as a metabolic disease	2010	Review	63	http://www.nutritionandmetabolism.com/content/7/1/7
Effects of curcumin on retinal oxidative stress and inflammation in diabetes	2007	Research	56	http://www.nutritionandmetabolism.com/content/4/1/8
The calorically restricted ketogenic diet, an effective alternative therapy for malignant brain cancer	2007	Research	52	http://www.nutritionandmetabolism.com/content/4/1/5
The effect of a low-carbohydrate, ketogenic diet versus a low-glycemic index diet on glycemic control in type 2 diabetes mellitus	2006	Research	48	http://www.nutritionandmetabolism.com/content/5/1/36
Curcumin and resveratrol inhibit nuclear factor-kappaB-mediated cytokine expression in adipocytes	2008	Research	45	http://www.nutritionandmetabolism.com/content/5/1/17
Dietary carbohydrate restriction in type 2 diabetes mellitus and metabolic syndrome: time for a critical appraisal	2008	Review	42	http://www.nutritionandmetabolism.com/content/5/1/9
Comparison of isocaloric very low carbohydrate/high saturated fat and high carbohydrate/low saturated fat diets on body composition and cardiovascular risk	2006	Research	41	http://www.nutritionandmetabolism.com/content/3/1/7
Effects of beta-hydroxy-beta-methylbutyrate (HMB) on exercise performance and body composition across varying levels of age, sex, and training experience: a review	2008	Review	35	http://www.nutritionandmetabolism.com/content/5/1/1

**Table 3:** Highly cited articles in Google Scholar published since 2006 (retrieved 30 January 2014)

**Title**	**Year published**	**Article type**	**Citations**	**Link**
Chocolate and prevention of cardiovascular disease: a systematic review	2006	Review	206	http://www.nutritionandmetabolism.com/content/3/1/2
Cancer as a metabolic disease	2010	Review	159	http://www.nutritionandmetabolism.com/content/7/1/7
Effects of curcumin on retinal oxidative stress and inflammation in diabetes	2007	Research	138	http://www.nutritionandmetabolism.com/content/4/1/8
Conjugated linoleic acids as functional food: an insight into their health benefits	2009	Review	100	http://www.nutritionandmetabolism.com/content/6/1/36
The calorically restricted ketogenic diet, an effective alternative therapy for malignant brain cancer	2007	Research	98	http://www.nutritionandmetabolism.com/content/4/1/5
The effect of a low-carbohydrate, ketogenic diet versus a low-glycemic index diet on glycemic control in type 2 diabetes mellitus	2008	Research	96	http://www.nutritionandmetabolism.com/content/5/1/36
Dietary carbohydrate restriction in type 2 diabetes mellitus and metabolic syndrome: time for a critical appraisal	2008	Review	93	http://www.nutritionandmetabolism.com/content/5/1/9
Effects of beta-hydroxy-beta-methylbutyrate (HMB) on exercise performance and body composition across varying levels of age, sex, and training experience: a review	2008	Review	88	http://www.nutritionandmetabolism.com/content/5/1/1
Curcumin and resveratrol inhibit nuclear factor-kappaB-mediated cytokine expression in adipocytes	2008	Research	87	http://www.nutritionandmetabolism.com/content/5/1/17

Further surprises came when we analyzed highly accessed articles. They were all review articles except for one commentary (Table 4), and only 50% of the highly accessed reviews were also highly cited according to Google Scholar (Table 1), supporting the conclusion that our articles are also being accessed regularly by individuals who are not actively involved in writing scientific articles: the general public. The three most highly accessed reviews cover the topics of low-carbohydrate diets, fructose and the impact of high protein on kidney function, respectively, and these subject areas were replicated throughout the top 10. They are clearly of interest to the general public; searching the article titles using the search engine Google revealed that all continue to be discussed and cited in online conversations, maintaining a steady stream of accesses and ensuring the articles continue to be referenced and explored long after they were published. We think this in part due to their open-access status. The benefits of open access are two-fold: encouraging public interest in the biology behind nutrition, and giving interested parties, including clinicians, access to peer-reviewed information on nutrition and diet.

**Table 4:** Highly accessed articles (retrieved 21 January 2014)

**Title**	**Year published**	**Article type**	**Online accesses**	**Link**
Ketogenic diets and physical performance	2004	Review	195075	http://www.nutritionandmetabolism.com/content/1/1/2
Fructose, insulin resistance, and metabolic dyslipidemia	2005	Review	179076	http://www.nutritionandmetabolism.com/content/2/1/5
Dietary protein intake and renal function	2005	Review	168257	http://www.nutritionandmetabolism.com/content/2/1/25
Diet induced thermogenesis	2004	Review	98261	http://www.nutritionandmetabolism.com/content/1/1/5
Effects of beta-hydroxy-beta-methylbutyrate (HMB) on exercise performance and body composition across varying levels of age, sex, and training experience: a review	2008	Review	88793	http://www.nutritionandmetabolism.com/content/5/1/1
Chocolate and prevention of cardiovascular disease: a systematic review	2006	Review	72872	http://www.nutritionandmetabolism.com/content/3/1/2
Cancer as a metabolic disease	2010	Review	69036	http://www.nutritionandmetabolism.com/content/7/1/7
A low-carbohydrate, ketogenic diet to treat type 2 diabetes	2005	Research	64754	http://www.nutritionandmetabolism.com/content/2/1/34
Very-low-carbohydrate diets and preservation of muscle mass	2006	Commentary	63580	http://www.nutritionandmetabolism.com/content/3/1/9
Dietary carbohydrate restriction in type 2 diabetes mellitus and metabolic syndrome: time for a critical appraisal	2008	Review	63361	http://www.nutritionandmetabolism.com/content/5/1/9

*Nutrition & Metabolism* strives to serve the academic and clinical communities and the public by publishing the highest quality research across all areas of nutrition in an accessible, open-access format. Ten years after we published our first article, the journal continues to expand and improve. In spite of the weaknesses highlighted above, we remain particularly proud that the journal Impact Factor has increased consistently since 2009. Beyond the simple matter of citations, *Nutrition & Metabolism* has always been a forum for controversial and groundbreaking studies, and we aim to encourage debate amongst our readership. We believe that publication is just the start of an article’s life, and greatly appreciate the numerous letters, commentaries and reviews we receive each year.

This evaluation indicates that *Nutrition & Metabolism* has published research and review articles across a broad range of subjects that are appreciated and cited by authors. Further, many of its articles are also of interest to the general public. We are gratified that *Nutrition & Metabolism* is responding to the interests of both the academic community and the public at large. With your support we will continue to provide this service to the best of our abilities.

